# Unveiling the cytotoxic potential of four *Callistemon* fruit extracts against breast and colon cancer: a combined metabolomic and in silico approach

**DOI:** 10.1186/s12906-025-05224-y

**Published:** 2026-01-15

**Authors:** Amira Y. Eissa, Kamilia F. Taha, Abeer Dahab, Usama R. Abdelmohsen, Khayreya A. Youssif, Mona H. Ibrahim, Seham S. El-Hawary, Manal M. Sabry

**Affiliations:** 1 Phytochemistry Department, Egyptian Drug Authority (EDA), Giza, Cairo 12556 Egypt; 2 Department of Medicinal and Aromatic Plants, Horticulture Research Institute- Giza, Cairo, 12211 Egypt; 3 Deraya Center for Scientific Research, Deraya University, New Minia, 61111 Egypt; 4 Pharmacognosy Department, Faculty of Pharmacy, Minia University, Minia, 61519 Egypt; 5 Department of Pharmacognosy, Faculty of Pharmacy, El Saleheya El Gadida University, El Sharquia, Egypt; 6 Department of Pharmaceutical Medicinal Chemistry and Drug Design, Faculty of Pharmacy for Girls, Al-Azhar University, Nasr City, Cairo Egypt; 7 Pharmacognosy Department, Faculty of Pharmacy, Cairo University, Cairo, 11562 Egypt

**Keywords:** Callistemon, Cancer cell lines, Cytotoxicity, CDK6, Molecular docking, Metabolomic, MTT assay, Flow cytometry, Apoptosis

## Abstract

**Background:**

Breast cancer and colon cancer are among the most prevalent malignancies worldwide, representing significant public health challenges. This study aimed to evaluate the potentially cytotoxic effect of fruit ethanol extracts of four selected *Callistemon* species: *Callistemon citrinus* (Curtis) Skeels, *Callistemon* *macropunctatus* (Dum.Cours.) Court, *Callistemon viminalis* (Sol. ex Gaertn) and *Callistemon subulatus* Cheel against breast (MCF-7) and colon (Caco-2) cancer cell lines in order to investigate the mechanism of action.

**Methods:**

metabolic profiling of the four selected *Callistemon* species was assessed using UPLC-ESI-MS/MS analysis. The in vitro cytotoxicity effects of the tested ethanol extracts against breast (MCF-7) and colon (Caco-2) carcinoma cell lines were assessed by means of 3-(4,5-dimethylthiazol-2- yl)-2,5-diphenyltetrazolium bromide (MTT) assay. The most active extract cell cycle analysis was subjected to flow cytometry. In-silico docking analysis of the most abundant metabolites against cell cycle regulatory enzymes was conducted, followed by molecular docking simulations for top binders.

**Results:**

Among the four tested *Callistemon* species, the extract derived from *C. macropunctatus* exhibited the most potent cytotoxic activity, with IC₅₀ values of 5.45 ± 0.34 µg/mL against MCF-7 breast cancer cells and 10.24 ± 0.59 µg/mL against Caco-2 colon cancer cells. These values indicate a higher cytotoxic potency compared to the reference drug staurosporine (IC₅₀ = 7.72 ± 0.46 µg/mL for MCF-7 and 5.16 ± 0.2 µg/mL for Caco-2). As a result, *C. macropunctatus* was selected for further analysis related to its ability to induce apoptosis and mechanistic effects. In total, sixteen compounds were tentatively identified, with flavonoids, lignans, and meroterpenes emerging as the dominant metabolites.Specifically, the extract caused S-phase arrest in MCF-7 breast cancer cells while both G0/G1 and S-phase arrest in case of Caco-2 colon cancer cells, indicating a broad-spectrum efficacy in disrupting cell cycle progression across different cancer types. To elucidate the underlying mechanisms, in-silico docking simulations were conducted to assess the binding affinities of the identified compounds towards CDK6, a critical regulator of the cell cycle. The evaluated compounds showed promising binding affinities ranging from − 6.5 to -9.7 kcal/mol, surpassing the binding efficiency of the co-crystal ligand of cyclin-dependent kinase (CDK6). Amongst the detected phenolic compounds, avicularin, nilocitin, and quercetin 3-*O*-(2’’-galloyl)-*β*-D-galactopyranoside exhibited the highest docking scores. These compounds formed strong interactions with essential amino acid residues in the CDK6 active site, suggesting a strong potential for inhibiting CDK6 activity.

**Conclusion:**

These findings warrant further exploration of *C. macropunctatus* extract as a promising anti-cancer agent, with a focus on elucidating its role of CDK6 inhibition and its antiproliferative effects.

**Supplementary Information:**

The online version contains supplementary material available at 10.1186/s12906-025-05224-y.

## Background

Cancer remains a major global health challenge, ranking second among the leading causes of death and accounting for nearly one in six fatalities worldwide [1]. In Egypt, it is likewise the second primary cause of death, responsible for approximately 13–14% of mortalities [2], while coronary heart disease remains the leading cause, accounting for 32.4% [3].

Among cancers, breast cancer is the most commonly diagnosed malignancy in women and a leading cause of cancer-related mortality worldwide [4]. Hormone receptor (HR)-positive breast cancer, which constitutes about 80% of all breast cancer cases, has been the focus of extensive research due to the availability of effective targeted endocrine therapies [5].

Colorectal cancer is another major health burden, ranking third in incidence and second in cancer-related mortality globally [1]. Its clinical challenge lies in its asymptomatic onset, often leading to late diagnosis and reduced treatment success [1]. In advanced stages, it frequently metastasizes to distant organs most notably the liver and lungs substantially lowering survival rates [6]. Even after potentially curative therapy, recurrence is common [7].

Chemotherapy remains a cornerstone of cancer treatment; however, its effectiveness is frequently limited by adverse effects and the emergence of intrinsic or acquired resistance [8]. Advances in targeted therapies have improved outcomes for HR-positive breast cancer, while colorectal cancer management often combines cytotoxic agents such as oxaliplatin and 5-fluorouracil with inhibitors of vascular endothelial growth factor (VEGF) and epidermal growth factor receptor (EGFR), both of which are key therapeutic targets [9, 10]. Nonetheless, resistance to treatment remains a major obstacle, underscoring the need for novel and more potent therapeutic strategies.

Cyclin-dependent kinases (CDKs) are central regulators of the cell cycle and have been widely investigated for their potential in personalized cancer therapy [10, 11]. The cell cycle consists of four sequential phases: G1 (first gap), S (DNA synthesis), G2 (second gap), and M (mitosis) with the G1/S checkpoint playing a critical role in determining whether a cell commits to DNA replication or enters quiescence (G0). Progression through this checkpoint is driven by Cyclin D/CDK4/6 and Cyclin E/CDK2 complexes, which phosphorylate the retinoblastoma (RB) protein, releasing the E2F transcription factor. E2F then activates S-phase gene expression, enabling DNA synthesis and cell cycle progression [12]. Dysregulation of the CDK–RB–E2F pathway promotes uncontrolled cell proliferation, making it an attractive therapeutic target in breast, colorectal, and other cancers [13].

Unsurprisingly, the US Food and Drug Administration (FDA) has approved the CDK4/6 inhibitors palbociclib and abemaciclib for the treatment of hormone receptor-positive breast cancer depending on the observation that CDK4 and 6 hyperactivation is involved. Additionally, CDK 6 mediates drug resistance in such cancer type treatment [14]. Furthermore, palbociclib was suggested as a positive candidate for colorectal cancer due to its CDK4/6 inhibitor activity [15] and is registered in several clinical trials (NCT03981614).

Natural products have played a pivotal role in drug discovery, with over 60% of current anticancer agents derived directly or indirectly from natural sources [16]. Plant extracts are particularly valuable due to their structural diversity, rich content of bioactive phytochemicals, and ability to modulate multiple signalling pathways, potentially improving efficacy and reducing toxicity compared to single-target agents [17–22]. Their multi-targeted actions, combined with a generally favourable safety profile, make them promising leads for novel therapeutic development [23, 24]. Importantly, many plant-derived compounds act synergistically with conventional therapies and help overcome drug resistance, making them especially relevant in breast and colorectal cancers, where treatment failure remains a major challenge [25].

The genus *Callistemon* (family Myrtaceae), commonly known as bottlebrush [26], is a rich source of structurally diverse metabolites, including triterpenoids, C-methylated flavonoids, phenolics, phloroglucinol derivatives, and essential oils. Traditionally, *Callistemon viminalis* has been used in Chinese folk medicine for treating haemorrhoids [27] and in agriculture for natural weed control [28]. Various species have demonstrated antimicrobial, antioxidant, antithrombotic, nematocidal, and insecticidal activities, largely attributed to their volatile oils and polyphenolic content [29]. Despite this broad biological potential, the anticancer properties of *Callistemon* remain largely unexplored, especially compared to well-studied medicinal plants such as *Curcuma longa* and *Camellia sinensis*, which have been extensively investigated in breast and colorectal cancers [30–33].

Notably, *Callistemon* is distinguished by its abundance of C-methylated flavonoids and phloroglucinol derivatives compounds rare in other genera which may confer unique anticancer mechanisms [22, 34, 35].

To address this gap, the present study provides the first comparative evaluation of ethanol fruit extracts from four *Callistemon* species (*C. citrinus*, *C. subulatus*, *C. macropunctatus*, and *C. viminalis*) against breast (MCF-7) and colon (Caco-2) carcinoma cell lines. In addition to cytotoxicity assessment, the study integrates cell cycle analysis, UPLC-ESI-MS/MS metabolite profiling, and molecular docking to investigate the interactions of abundant metabolites with cell cycle–regulating enzymes. This multi-pronged approach aims to highlight the underexplored anticancer potential of *Callistemon* and provide mechanistic insights that distinguish it from more extensively studied medicinal plants, positioning this genus as a promising source of novel therapeutic candidates.

## Materials and methods

### Plant material

Fruits of *Callistemon citrinus* (Curtis) Skeels, *Callistemon macropunctatus* (Dum.Cours.) Court, *Callistemon viminalis* (Sol. ex Gaertn.), and *Callistemon subulatus* Cheel were collected from the public “El-Orman Garden” (coordinates: 30°01′45″ N, 31°12′47″ E; Giza, Egypt) during the spring season of 2022 when capsules were fully mature (dry and woody), ensuring sufficient material for extraction. Collection was performed in accordance with the regulations of the garden and the official scientific plant collection guidelines of Egypt. Plant species were authenticated by Engineer Thereas Labib, Senior Botanist and Head Specialist for Plant Identification at El-Orman Botanical Garden, Giza, Egypt. Voucher specimens (Nos. PN-08–11−2019 A, PN-08–11−2019B, PN-08–11−2019 C, and PN-08–11−2019D, respectively) were deposited in the Museum of the Pharmacognosy Department, Faculty of Pharmacy, Cairo University.

### Chemicals

Solvents of analytical grade utilized for the extraction procedures were obtained from El- Gomhouria for Drugs Co. (Cairo, Egypt), While HPLC grade solvents (ethanol, acetonitrile) were purchased from Thermo Fisher Scientific UK Ltd. (Loughborough, Leicestershire, UK); Purification of water was done applying a Milli-Q system (Millipore, Bedford, MA, USA). Kit for the in vitro cytotoxic assay (MTT Based) was obtained from (Sigma-Aldrich, St. Louis, MO, USA; Catalog Nos. M5655 and M8910).

### Preparation of the extracts

Fruits of each of the four *Callistemon* species were collected and air-dried at room temperature (25 ± 2 °C) for 15 days in a well-ventilated, shaded area, protected from direct sunlight to prevent photodegradation of sensitive metabolites, until completely dry. The ambient relative humidity was approximately 40–50%, ensuring gradual drying without microbial growth. The dried fruits were then ground into a fine powder and stored in sealed amber glass bottles. A 25 g portion of the powdered fruits from each species was extracted four times with 20 mL of 90% ethanol by cold maceration until exhaustion. The combined ethanolic extracts of each species were filtered and concentrated under reduced pressure using a rotary evaporator (Heidolph, Germany) at a temperature not exceeding 40 °C. Yields ranged from 1.9 to 2.1 g (7.6–8.4% w/w), depending on the species.The obtained solvent-free residues were stored at 4 °C until they were subjected to Ultra-Performance Liquid Chromatography coupled with Electrospray Ionization Tandem Mass Spectrometry (UPLC-ESI-MS/MS) for phytochemical analysis. The dried ethanolic extracts of each *Callistemon* species were reconstituted in methanol to obtain a stock solution of 1 mg/mL. The reconstituted extracts were filtered through a 0.22 μm syringe filter to ensure clarity and sterility. Stock solutions were stored in amber glass vials at − 20 °C until UPLC-ESI-MS/MS analysis to prevent light and temperature induced degradation. For in vitro assay, the dried ethanolic extracts of *Callistemon* species were reconstituted in dimethyl sulfoxide (DMSO) to prepare a stock solution of 10 mg/mL. The stock was aliquoted and stored at − 20 °C in amber vials to prevent repeated freeze–thaw cycles and light-induced degradation. For the MTT cytotoxicity assay and Annexin V-FITC apoptosis assay, the stock solution was diluted with complete culture medium to the desired working concentrations, ensuring that the final DMSO concentration did not exceed 0.1% (v/v) to avoid solvent-related cytotoxicity. Fresh working solutions were prepared immediately prior to each experiment to maintain extract stability.

### Metabolomic profiling

Ethanolic extracts of *C. citrinus; C. subulatus; C. macropunctatus* and *C. viminalis* were subjected to liquid chromatographic analysis [36–38] on an Acquity (UPLC-ESI-MS) system coupled to a quadrupole time-of- flight hybrid mass spectrometer (Synapt G2 HDMS, Waters Milford, USA). Chromatographic elution was performed on an ethylene Bridged Hybrid Technology using C_18_ column (2.1 × 100 mm, with particle size of 1.7 μm: Waters Milford, USA), the guard column with dimensions 2.1 × 5 mm, and 1.7 μm particle size. Linear binary solvent gradient was applied from 0 to 100% eluent B (acetonitrile) to eluent A (0.1% formic acid in water (v/v) over 6 min at a flow rate of 0.3 mL/min. 2 µL was injected the tray temperature was maintained at 12 °C and the column temperature was kept at 40^o^ C. High-resolution mass spectrometry was carried out in both positive and negative ESI ionisation modes with a spray capillary voltage of 4.5 kV, a capillary temperature of 320 °C, a cone voltage of 30 V, a desolvation gas temperature of 400 °C and a desolvation gas flow of 800 L/H. The mass range was acquired from *m/z* 150 to 1500.

Raw data were initially sliced into two data sets based on the ionization mode, using the MassConvert tool from ProteoWizard. The sliced data sets were imported into MZmine2.10; a framework for differential analysis of mass spectrometry data. The high-resolution mass spectral (MS1) data set was deconvoluted and deisotoped, while mass adducts, fragments, as well as complexes were primarily identified and sorted with MZmine 2.10. The spectra were crop-filtered from 2 to 35 min. The peaks in the samples and blanks were detected using the chromatogram builder. Mass ion. peaks were isolated with a centroid detector threshold that was greater than the noise level set to 1.0E4 and an MS level of 1. Following this, the chromatogram builder was used with a minimum time span set to 0.2 min, and the minimum height and m/z tolerance to 1.0E4 and 0.001 m/z or 5.0 ppm, respectively. Chromatogram deconvolution was then performed to detect the individual peaks. The local minimum search algorithm (chromatographic threshold: 90%, search minimum in RT range: 0.4 min, minimum relative height: 5%, minimum absolute height: 3.0E4, minimum ratio of peak top/edge: 2, and peak duration range: 0.3–5 min) was applied. Isotopes were also identified using the isotopic peaks grouper (m/z tolerance: 0.001 m/z or 5.0 ppm, retention time tolerance: 0.2 absolute (min), maximum charge: 2, and representative isotope: most intense). The retention time normalizer (m/z tolerance: 0.001 m/z or 5.0 ppm, retention time tolerance: 5.0 absolute (min), and minimum standard intensity: 5.0E3) was used to reduce inter-batch variation. The peak lists were all aligned using the join aligner parameters set to m/z tolerance: 0.001 m/z or 5.0 ppm, weight for m/z: 20, retention time tolerance: 5.0 relative (%), weight for RT: 20. Missing peaks were detected using the gap filling peak finder (intensity tolerance: 1.0%, m/z tolerance: 0.001 m/z or 5.0 ppm, and retention time tolerance of 0.5 absolute (min)). An adduct search was performed for Na-H, K-H, NH4, formate, and ACN + H (RT tolerance: 0.2 absolute (min), m/z tolerance: 0.001 m/z or 5.0 ppm, max relative adduct peak height: 30%). Additionally, a complex search was performed (ionization method: [M + H] + for ESI positive mode and [M − H] − for ESI negative mode, retention time tolerance: 0.2 absolute (min), m/z tolerance: 0.001 m/z or 5.0 ppm, and with maximum complex peak height of 50%).

The Dictionary of Natural Products and METLIN were used as databases for the identification of secondary metabolites.

### Cell lines and culture conditions

Human breast adenocarcinoma (MCF-7, ATCC^®^ HTB-22™) and human colorectal adenocarcinoma (Caco-2, ATCC^®^ HTB-37™) cell lines were purchased from American Type Culture Collection (University Boulevard Manassas, Virginia, USA). Cell lines were authenticated by short tandem repeat (STR) profiling prior to experimental use and were routinely tested to confirm absence of Mycoplasma contamination. MCF-7 cells were cultured in DMEM (Dulbecco’s Modified Eagle Medium) (Invitrogen/Life Technologies, California, USA) supplemented with 10% heat-inactivated fetal bovine serum (FBS; Hyclone, USA), 10 µg/mL insulin (Sigma-Aldrich, St. Louis, MO, USA), 1% penicillin and 1% streptomycin (Sigma-Aldrich, St. Louis, MO, USA), whereas Caco-2 cells were maintained in DMEM with 10% heat-inactivated FBS and 1% penicillin, 1% streptomycin, without insulin supplementation. Cells were incubated at 37 ^◦^C in humidified 5% (v/v) CO_2_ atmosphere. The antiproliferative effects of the four ethanol extracts of *Callistemon* fruits were evaluated in 3 days post-treatment using different extract concentrations ranging from 0.4 to 100 µg/mL.

### Cytotoxicity assay (MTT Assay)

The in vitro cytotoxic effects of the tested ethanolic extracts were evaluated using the 3-(4,5-dimethylthiazol-2-yl)−2,5-diphenyltetrazolium bromide (MTT) assay, following the manufacturer’s instructions for the in vitro toxicology assay kit (Sigma-Aldrich, St. Louis, MO, USA). This colorimetric assay measures the metabolic activity of viable cells through the mitochondrial reduction of MTT salt (0.5 mg/mL final concentration) into insoluble formazan crystals. In this study, MCF-7 and Caco-2 cells were seeded at 1.2 × 10⁴ cells/well and 1.8 × 10⁴ cells/well, respectively, in 96-well plates to achieve 70–80% confluency after 24 h. Subsequently, 100 µL of the tested extract at concentrations ranging from 0.4 to 100 µg/mL was added, and the cells were incubated for 24 h. After incubation, MTT solution (0.5 mg/mL) was added to each well and plates were typically incubated for 3–4 h at 37 °C in the dark until formazan crystals were formed, followed by the addition of the solubilization solution (DMSO 100%) to dissolve the crystals completely. Absorbance was measured at 570 nm with a background correction at 690 nm using a Bio-line ELISA reader (USA) [39]. The experiment was conducted in triplicate for each concentration, and the concentration of each extract that resulted in 50% inhibition of cell viability (IC50) was determined using dose-response curve fit in GraphPad Prism software. Untreated cells served as negative controls, while staurosporine (5 µM) was used as a positive control for cytotoxicity.

### Flow cytometric analysis

The fruit ethanolic extract of *Callistemon macropunctatus* was selected for further flow cytometric analysis to evaluate its effects on cell cycle distribution and apoptosis induction in MCF-7 and Caco-2 cells.

### Cell cycle analysis (PI staining)

MCF-7 and Caco-2 cells were seeded in 6-well plates at 1 × 10^6^ cells/well, incubated overnight to allow adherence, and then treated with the IC₅₀ concentrations determined from the MTT assay for 24 h. Following treatment, both floating and adherent cells were collected, washed twice with ice-cold phosphate-buffered saline (PBS; pH 7.4), and fixed by the dropwise addition of 2 mL of precooled 66% ethanol with gentle shaking. The cells were incubated at 4 °C for at least 2 h, washed twice with PBS, and resuspended in 1 mL of PBS containing 50 µg/mL RNase A and 10 µg/mL propidium iodide (PI; P4864, Sigma-Aldrich, St. Louis, MO, USA). To minimize clumping, the cell suspension was gently vortexed. Samples were incubated for 20 min at room temperature in the dark. Analysis of the DNA content was performed using phycoerythrin emission signal detector (FL2) at 535/617 nm (BD FACSCalibur™, flow cytometer, Biosciences, USA) acquiring at least 10,000 single cell events per sample and gating out debris and doublets. The distribution of cell cycle stages was calculated using FlowJo software (USA, version 10.7.1) [40].

### Apoptosis analysis (Annexin V-FITC/PI Staining)

Apoptosis was evaluated using the Annexin V-FITC/PI Apoptosis Detection Kit (Catalog: K101-25), (BioVision, Mountain View, CA, USA) following the manufacturer’s instructions. After 24 h of treatment with IC₅₀ concentrations, both floating and adherent cells were harvested, washed twice with cold PBS, and resuspended in 1× binding buffer at 1 × 10⁶ cells/mL. Then, 5 µL of Annexin V-FITC and 5 µL of PI (50 µg/mL) were added to 100 µL of cell suspension, followed by 15 min of incubation in the dark at room temperature. Finally, 400 µL of binding buffer was added, and samples were immediately analysed by flow cytometry (BD FACSCalibur™) using FL1 (FITC) at 535 nm and FL2 at 617 nm (PI) channels.

Untreated cells used as negative control for both cell cycle and apoptosis.

### Statistical analysis

Experimental results were calculated from three replicates using GraphPad Prism v.8.0 (San Diego, CA, USA). IC_50_ was determined from dose-response best fit curve. Data are presented as mean ± standard deviation (SD). Statistical significance was determined at *p* < 0.05 applying Student’s *t*-test or one way ANOVA followed by Tukey’s HSD for multiple comparisons. Microsoft Excel was used for the graphical presentation of data.

### Docking study and pharmacokinetics estimates

AutoDock 4.2 was employed to conduct molecular docking of 16 compounds against CDK6 (PDB: 4tth) [41]. The PDB code of the crystallographic structure of the protein was downloaded from the Protein Data Bank, then the compounds were drawn using https://www.rcsb.org/chemical-sketch website and saved as pdb file extension. Following established protocols, ligand and protein files, as well as grid, and docking parameter files were prepared [42]. The crystallographic structures of the protein were obtained from the protein database. A 3D grid box with dimensions of 60 × 6 × 6 Å (x, y, z) was used for docking into CDK6, centered at coordinates − 32.56, 24.37, and − 13.83 Å, with a grid spacing of 0.375 Å [43, 44].

The docking results were analyzed and visualized using BIOVIA Discovery Studio Visualizer. The compounds need to be investigated for their pharmacokinetic properties. So, the ADMET was assessed computationally using the SwissADME server, and Datawarrior program for the highest docking score compounds (avicularin, nilocitin, and quercetin3-O-(2’’-galloyl)-beta-D galactopyranoside) (Table S1).

## Results and discussion

**Fig. 1:**
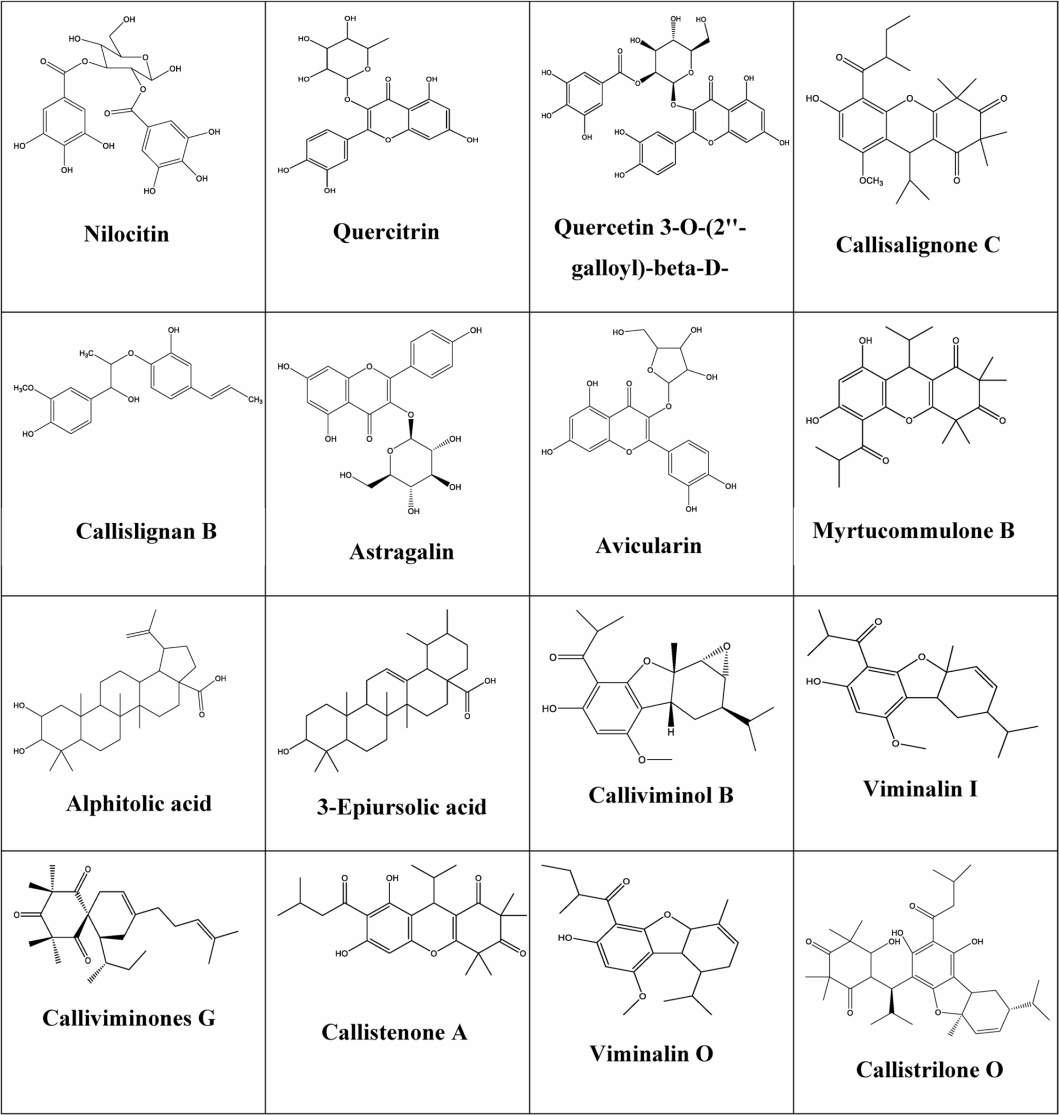
Identified compounds from the fruit ethanol extracts of *Callistemon* species

Metabolic profiling UPLC-ESI-MS/MS analysis tentatively identified 16 compounds within the *Callistemon* species fruit ethanol extracts (Supplementary Figures S1-S4) and Table 1.. Dereplication of the phytoconstituents from the ethanolic extract of *C. citrinus*, *C. subulatus*, *C. macropunctatus*, and *C. viminalis* lead to the detection of phenolics, flavonols, lignans, terpenes, *β*-Triketones, and acylphloroglucinol derivatives (Table 1.; Fig. 1).

**Table 1:** Identified metabolites in the fruit ethanolic extract of selected *Callistemon* species

No	m/z	R.T. (min)	M. wt.	Compounds Identified	**Chemical class**	**Molecular formula**	**Mass error**	**Source**	**MS/MS fragmentation**	**References**
1	485.09342	2.79	484.08614	Nilocitin	Phenolic acid	C_20_H_20_O_14_	0.00083	*C. viridifloro*	315.1, 317.1, 299.1, 297.15	[35]
2	449.10843	3.18	448.10115	Quercitrin	Flavonol	C_21_H_20_O_11_	0.000586	*C. viminalis*	403.19, 375.21	[36]
3	617.11489	8.04	616.10761	Quercetin 3-O-(2''-galloyl)-β-D-galactopyranoside	Flavonol	C_28_H_24_O_16_	0.00117	*C. citrinus*	464.2, 303.04	[55]
4	444.24478	8.04	443.2375	Callisalignone C	Acylphloroglucinol	C_26_H_35_O_6_	0.005865	*C. salignus C. viminalis*	401.15, 393.18, 391.2, 371.14	[48]
5	331.1543	8.54	330.14703	Callislignan B	Lignan	C_19_H_22_O_5_	0.000305	*C. citrinus*	329.0054, 313.1075, 301.1076, 285.1335	[46]
6	449.1085	8.70	448.10122	Astragalin	Flavonol	C_21_H_20_O_11_	0.000656	*C. citrinus*	287.09	[37]
7	435.09294	8.89	434.08566	Avicularin	flavonol	C_20_H_18_O_11_	0.000745	*C. viminalis*	391.28, 361.13	[53]
8	415.21185	17.97	414.20457	Myrtucommulone B	β-triketones	C_24_H_30_O_6_	0.00033	*C. citrinus* *C. rigidus*	397.32, 371,31, 259.16	[47]
9	473.3635	21.17	472.35622	Alphitolic acid	Triterpenoid	C_30_H_48_O_4_	0.00004	*C. citrinus*	437.39, 387.25	[39]
10	457.3684	21.53	456.36112	3-Epiursolic acid	Triterpenoid	C_30_H_48_O_3_	0.000775	*C. citrinus*	439.35, 411.32, 393.26,	[38]
11	361.20107	21.60	360.19379	Calliviminol B	Acylphloroglucinol	C_21_H_28_O_5_	0.000115	*C. viminalis*	343.19, 299.25, 291.25	[50]
12	345.20617	22.75	344.19889	Viminalin I	Acylphloroglucinol	C_21_H_28_O_4_	0.00013	*C. viminalis*	329.2, 327.12, 287.23, 275.2007	[49]
13	387.29362	23.05	386.28634	Calliviminones G	Acylphloroglucinol	C_25_H_38_O_3_	0.004245	*C. viminalis*	331.16, 329.25, 313.1068, 261.185	[51]
14	429.2274	23.35	428.22012	Callistenone A	Acylphloroglucinol	C_25_H_32_O_6_	0.00023	*C. viminalis*	357.205, 331.26, 329.25, 327.1227	[52]
15	359.22219	23.55	358.21491	Viminalin O	Acylphloroglucinol	C_22_H_30_O_4_	0.0005	*C. viminalis*	307. 154, 301.21, 287.23, 259. 20	[56]
16	583.34818	23.90	582.3409	Callistrilone O	Meroterpenoid	C_35_H_48_O_7_	0.000895	*C. citrinus*	567.33, 527.43, 239.14	[57]

Phenolic acids: One phenolic acid: nilocitin (Peak 1), having a molecular ion peak at *m/z* 485.09342 [M + H]^+^ (RT, 2.79 min) C_20_H_20_O_14_, was previously reported in *Callistemon viridiflora* [35].

Flavonoids: Four flavonol derivatives were identified in the four *Callistemon* fruit extracts under study (Table 1.). Peaks 2, 3 and 7 were assigned as quercetin derivatives, with molecular formulae C_21_H_20_O_11_, C_28_H_24_O_16_ and C_20_H_18_O_11_ and molecular ion peaks at *m/z* 449.10843 [M + H]^+^ (RT, 3.18 min), *m/z* 617.11489 [M + H]^+^ (RT, 8.04 min) and *m/z* 435.09294 [M + H]^+^ (RT, 8.89), identified as quercitrin (Peak 2), Quercetin 3-*O*-(2’’-galloyl)-β-D-galactopyranoside (Peak 3), and avicularin (peak 7), respectiveley. Furthermore, peak 6 was assigned for kaempferol glycoside (astragalin) exhibiting a molecular formula C_21_H_20_O_11_ and *m/z* 449.1085 [M + H]^+^ (RT, 8.70). In agreement with the reported data, these flavonols were previously extracted from *Callistemon citrinus* [37, 45].

Lignans: Following the chemical formulae C_19_H_22_O_5_, the mass ion peaks at *m/z* 331.1543[M + H]^+^ (RT, 8.54 min) were recognized as callislignan B (Peak 5), which were previously isolated from *Callistemon citrinus* [46].

*β*-Triketones: Myrtucommulone B (peak8) was identified from the mass ion peaks at *m/z* 415.21185 [M + H]^+^ (RT, 17.97 min) C_24_H_30_O_6_. It was extracted from *Callistemon citrinus*,* Callistemon rigidus*,* Callistemon salignus*, and *Callistemon viminalis*, respectively [47, 48].

Acylphloroglucinol: Six acylphloroglucinol were detected in the four selected fruit extracts. The molecular formulas C_26_H_35_O_6_, C_21_H_28_O_5,_ C_21_H_28_O_4,_ C_25_H_38_O_3,_ C_25_H_32_O_6,_ and C_22_H_30_O_4_, which were anticipated, and identified as callisalignone C (peak 4), calliviminol B (peak 11), viminalin I (peak 12), calliviminones G (peak 13), callistenone A (peak 14), and viminalin O (peak 15), based on their protonated molecular ion peaks [M + H]^+^ at *m/z* 444.24478 (RT 8.04), *m/z* 361.20107 (RT, 21.60 min), *m/z* 345.20617 (RT, 22.75 min), *m/z* 387.29362 (RT, 23.05 min), *m/z* 429.2274 (RT, 23.35 min), *m/z* 359.22219 (RT, 23.55 min), which previously isolated from *Callistemon viminalis* [49–53].

Terpenes: Two triterpenes were identified in the four selected fruit extracts as alphitolic acid (peak 9) and 3-epiursolic acid (peak 10), based on their chemical formula C_30_H_48_O_4_ and C_30_H_48_O_3_, with molecular ion peaks [M + H] ^+^ at *m/z* 473.3635 (RT, 21.17 min) and 457.3684 (RT, 21.53 min), respectively. They were previously reported in *C. citrinus and C. lanceolatus* [38, 39, 54]. Furthermore, one meroterpene was.

assigned to callistrilone O with molecular ion peak [M + H] ^+^ at *m/z* 583.34818 (RT, 23.90 min). It was previously reported in *C. citrinus* [22].

**Fig. 2:**
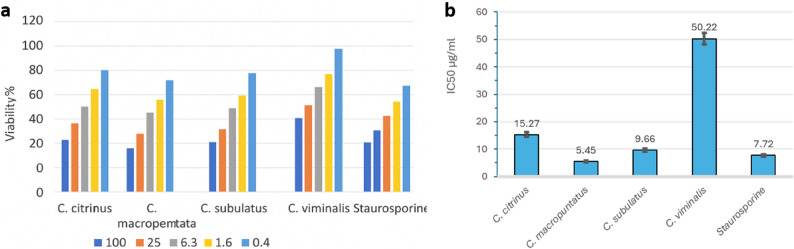
**a** Concentration-dependent antiproliferative effect of the four *Callistemons* fruit extracts on MCF-7 cell line using MTT assay. **b** IC_50_ values (µg/mL) for the four extracts. Data are reported as mean values ± SD for triplicates

**Fig. 3:**
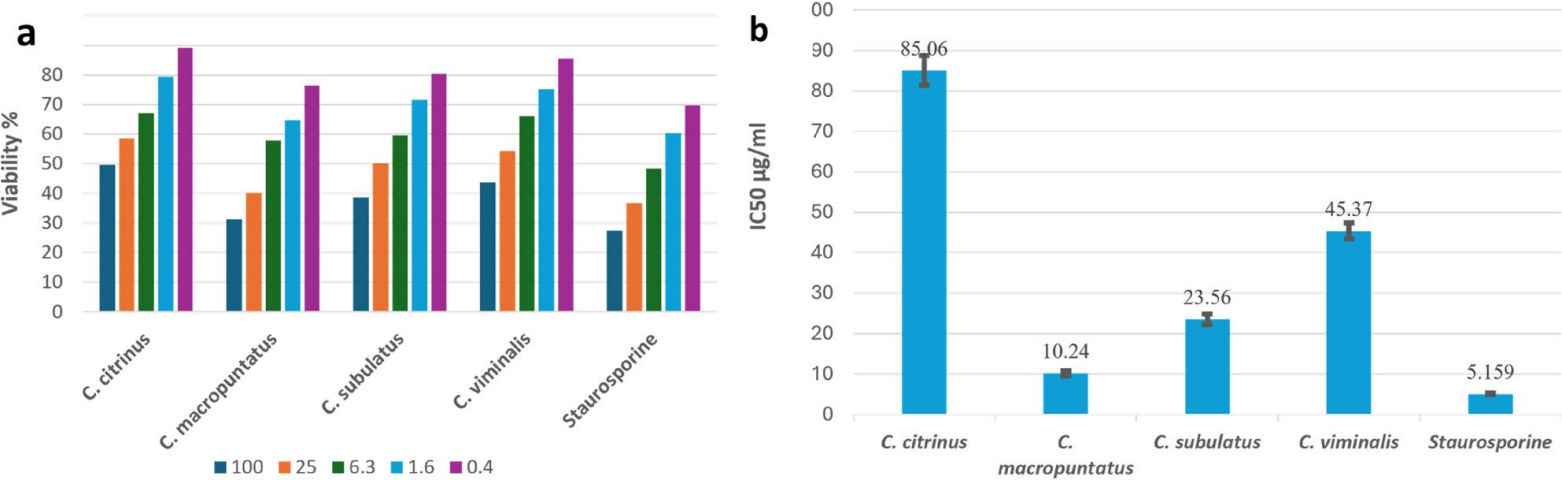
**a** Concentration-dependent antiproliferative effect of the four *Callistemons* fruit extracts on Caco-2 cell line using MTT assay. **b** IC_50_ values (µg/mL) for the four extracts. Data are reported as mean values ± SD for triplicates

The ethanol fruit extracts of the four *Callistemon* species demonstrated concentration-dependent antiproliferative effects against the MCF-7 and Caco-2 cancer cell lines (Figs. 2A and 3A). Among the tested extracts, *C. macropunctatus* exhibited the most potent cytotoxic activity, with IC₅₀ values of 5.45 ± 0.34 µg/mL for MCF-7 and 10.24 ± 0.59 µg/mL for Caco-2 as shown in Table 2; Figs. 2B and 3B. With an IC_50_ value of 9.66 ± 0.59 and 23.56 ± 1.28 µg/mL, respectively, the ethanol extract of *C*. *subulatus* shown excellent efficacy against MCF-7 and Caco-2. In contrast, *C. viminalis* displayed the weakest activity against MCF-7 with IC₅₀ (50.22 ± 2.06 µg/mL), while *C. citrinus* was the least active against Caco-2 with IC₅₀ (85.06 ± 3.61 µg/mL). For comparison, the reference drug staurosporine exhibited IC₅₀ values of 7.72 ± 0.46 µg/mL for MCF-7 and 5.16 ± 0.20 µg/mL for Caco-2. The *C. macropunctatus* extract demonstrated greater cytotoxic potency against MCF-7 cells, with an IC₅₀ approximately 1.4-fold lower than that of staurosporine (5.45 ± 0.34 µg/mL vs. 7.72 ± 0.46 µg/mL). In contrast, its potency against Caco-2 cells was lower than staurosporine, with an IC₅₀ of 10.24 ± 0.59 µg/mL compared to 5.16 ± 0.20 µg/mL.Overall, the data confirm that the ethanol fruit extract of *C. macropunctatus* possesses the most promising cytotoxic activity among the tested *Callistemon* species.

**Table 2:** Cytotoxic results (IC_50_) of four *Callistemons* fruit extracts against MCF-7 and Caco-2 cell lines

Plant name	Cytotoxicity * (IC_50_ µg/mL)	
	MCF-7	Caco-2
*C. citrinus*	15.27 ± 0.87^a^	85.06 ± 3.61 ^a^
*C. macropunctatus*	5.45 ± 0.34^b^	10.24 ± 0.59 ^b^
*C. subulatus*	9.66 ± 0.59^c^	23.56 ± 1.28 ^c^
*C. viminalis*	50.22 ± 2.06^d^	45.37 ± 1.99 ^d^
Staurosporine	7.72 ± 0.46	5.16 ± 0.2

* Results are the mean of three independent tests ± SD. Statistical analysis was performed using One way ANOVA followed by Tukey post-hoc test, letters indicate significance at p < 0.05.

**Fig. 4:**
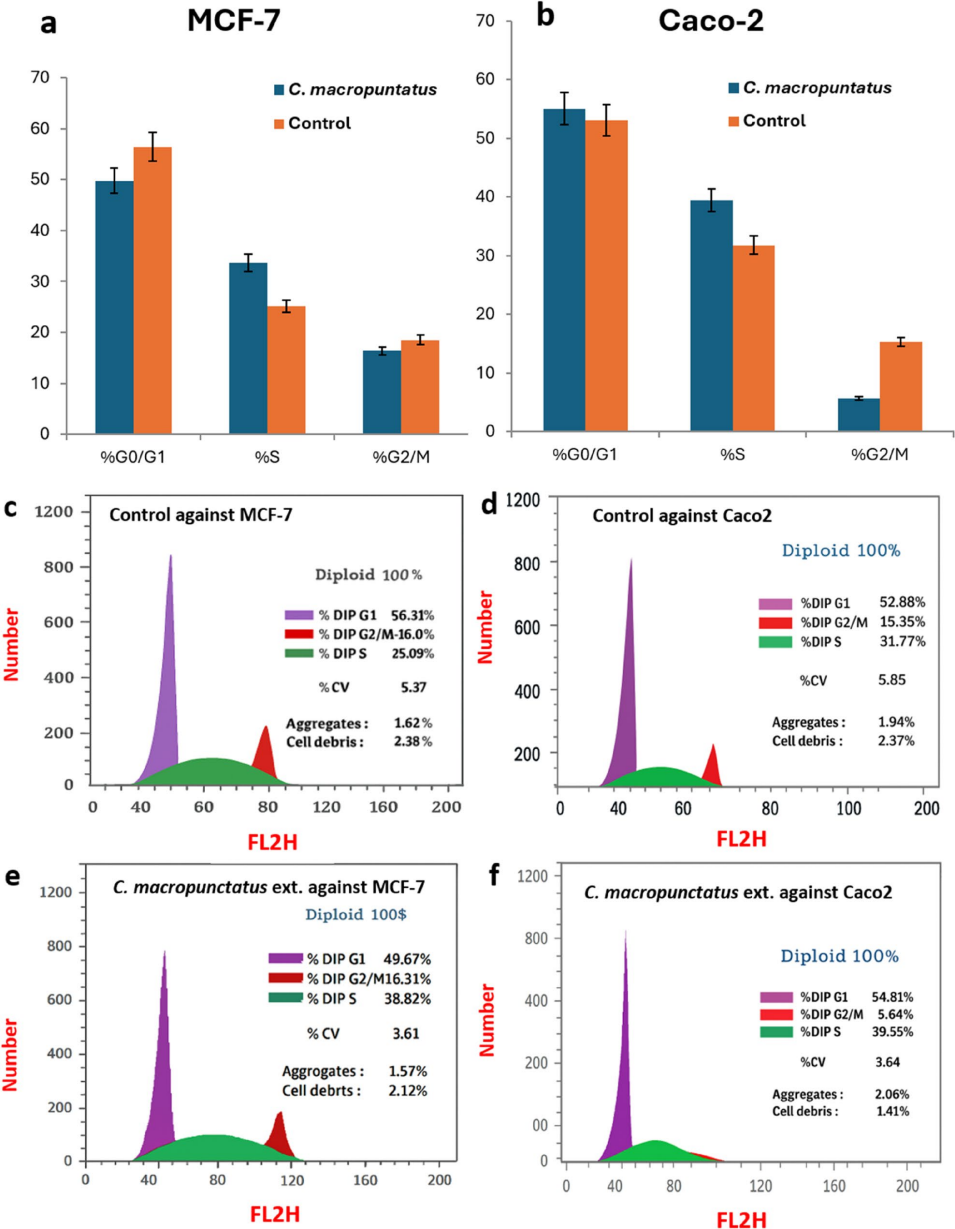
Effects of *C. macropunctatus* fruit extract on the distribution of the various cell cycle phases in MCF-7 cell line or CACO-2 cell line. (**a)** histogram of MCF-7, (**b**) histogram of Caco-2, (**c**) control against MCF-7, (**d**) control against Caco-2, (**e**) *C. maropunctatus* against MCF-7, (f) *C. maropunctatus* against Caco-2. Histograms represent data as mean ± SD. *n* = 3 independent experiments; Student’s t-test was used for statistical analysis and * indicates statistically significant against control on each stage at *p* < 0.05

Accordingly, the *C. macropunctatus* fruit extract may provide chemotherapeutic or chemoprotective isolates. Thus, an assessment was conducted on the chemical composition of the *C. macropunctatus* extract as well as its potential antiproliferative mechanism. As a result, the *C*. *macropunctatus* ethanol extract was chosen for additional examination of cell cycle distribution. As the inhibition of cell cycle progression is a key anticancer strategy, flow cytometry was utilized to assess the possible antiproliferative mechanism of the *C. macropunctatus* extract to determine disruption in MCF-7 and Caco-2 cell cycle distribution [58]. Flow cytometric analysis of DNA content stained with propidium iodide (PI) revealed that the fruit ethanolic extract of *Callistemon macropunctatus* significantly altered the cell cycle distribution of MCF-7 and Caco-2 cells. After treating MCF-7 cells with the extract at an IC_50_ concentration (5.45 µg/mL), the G0/G1 population decreased from 56.31 ± 1.01% in control cells to 49.67 ± 0.88% following treatment, while the S-phase population increased markedly from 25.09 ± 0.43% to 33.82 ± 1.11%. The G2/M population showed a slight decrease from 18.60 ± 0.63% to 16.51 ± 1.18%, suggesting that the extract induced a prominent S-phase arrest in MCF-7 cells, thereby inhibiting DNA replication and cell proliferation (Fig. 4).

Moreover, in the Caco-2, the extract induced a dual arrest at G0/G1 and S phases. The G0/G1 population increased slightly from 52.88 ± 1.11% to 54.81 ± 0.97%, while the S-phase population increased significantly from 31.77 ± 0.84% to 39.55 ± 0.64%. Concurrently, the G2/M phase decreased from 15.35 ± 0.43% to 5.64 ± 0.35%, indicating a reduction in cells entering mitosis (Fig. 4). These results demonstrate that *C. macropunctatus* fruit extract inhibits cell proliferation by inducing cell cycle arrest, which is a common anticancer mechanism of polyphenol- and terpenoid-rich plant extracts.

Results of flow cytometry and cell cycle inhibition stages are summarized in Table 3.

**Table 3:** Flow cytometry of *C. macropunctatus* fruit ethanol extract and its impact on MCF-7 and Caco-2 cell cycle inhibitory stages

Cell line		%G0/G1	%S	%G2/M	Comment
MCF7	*C. macropunctatus*	49.67 ± 0.88*	33.82 ± 1.11*	16.51 ± 1.18*	cell growth arrest at S phase
	Control	56.31 ± 1.01	25.09 ± 0.43	18.6 ± 0.63	---
Caco2	*C. macropunctatus*	54.81 ± 0.97	39.55 ± 0.64*	5.64 ± 0.35*	cell growth arrest at both G0/G1 and S phases
	Control	52.88 ± 1.11	31.77 ± 0.84	15.35 ± 0.43	---

Results are the mean of three independent tests ± SD. Statistical analysis was performed using Ostudent’s t- test. *Significance is considered at p < 0.05 against control at each phase

**Fig. 5:**
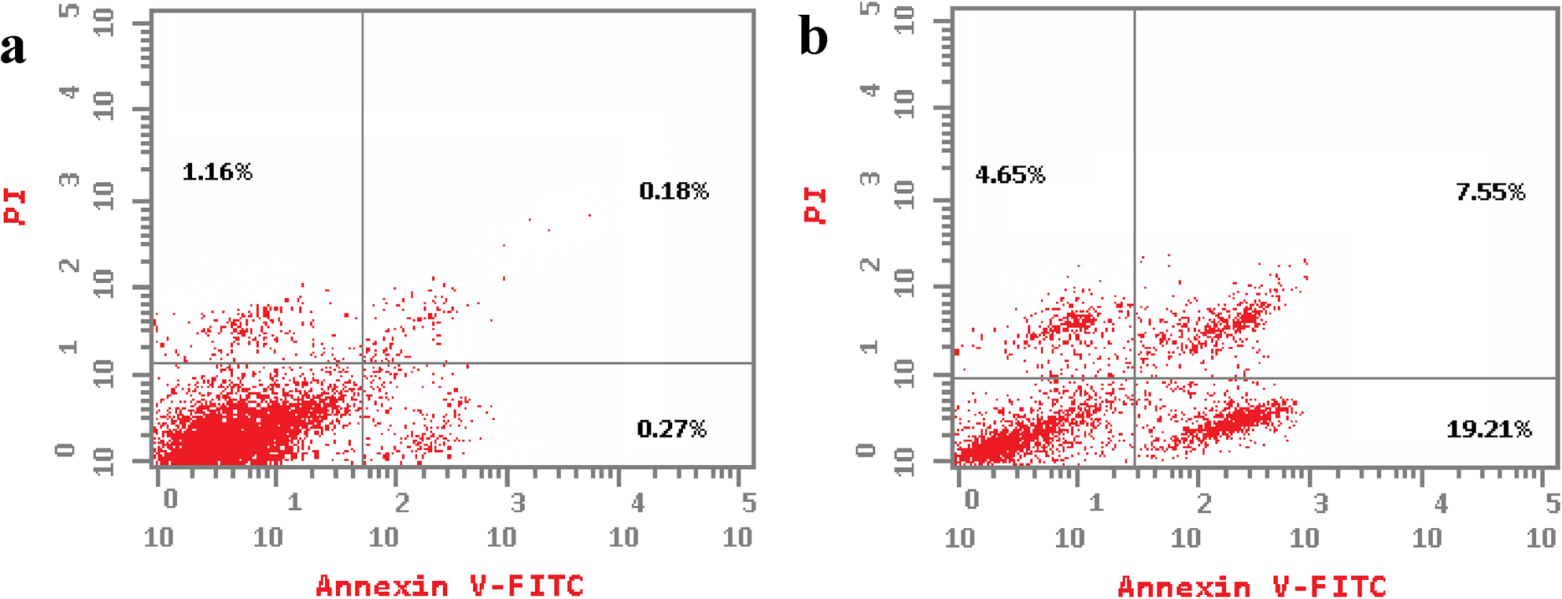
Annexin V-FITC/PI flow cytometric analysis of apoptosis and necrosis in MCF-7 cells after treatment with control treatment (**a**) and *C. macropunctatus* fruit ethanolic extract (**b**) for 24 h

**Fig. 6:**
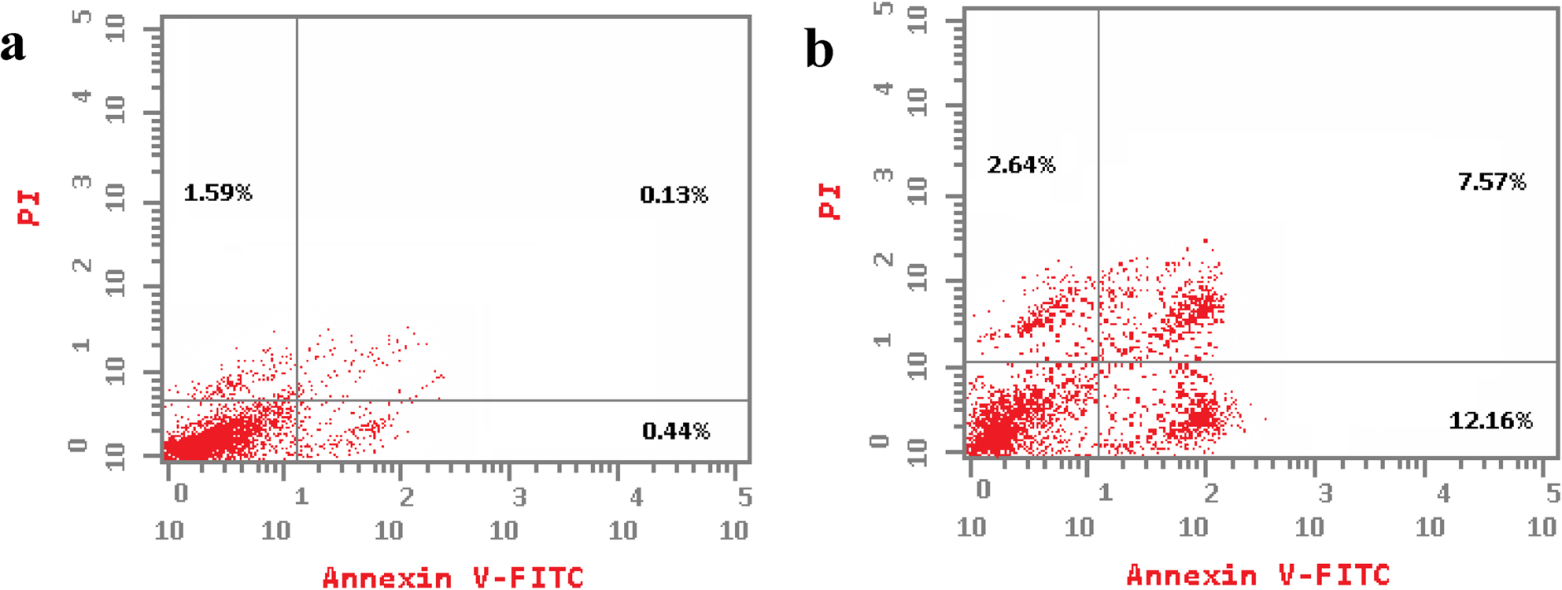
Annexin V-FITC/PI flow cytometric analysis of apoptosis and necrosis in Caco-2 cells after treatment with control treatment (**a**) and *C. macropunctatus* fruit ethanolic extract (**b**) for 24 h

Flow cytometric analysis using Annexin V-FITC/PI staining revealed that the ethanolic fruit extract of *Callistemon macropunctatus* significantly induced apoptosis in both MCF-7 and Caco-2 cells compared with the untreated controls (Table 4). In MCF-7 cells, the total apoptotic population increased from 1.61 ± 0.1% in the control to 31.41 ± 1.87% after treatment. Early apoptosis was the predominant stage (19.21 ± 1.35%), followed by late apoptosis (7.55 ± 0.62%), while necrotic cells accounted for 4.65 ± 0.35% (Fig. 5). Similarly, in Caco-2 cells, the extract increased total apoptosis from 2.16 ± 0.18% in the control to 22.37 ± 0.16% in the treated cells, with 12.16 ± 0.96% in early apoptosis, 7.57 ± 0.57% in late apoptosis, and 2.64 ± 0.21% necrosis (Fig. 6). These findings indicate that the extract primarily triggers apoptotic cell death rather than necrosis in both cancer cell lines. The pronounced increase in early apoptotic populations suggests that the extract activates programmed cell death pathways, potentially through mitochondrial-mediated apoptosis or caspase activation, as commonly observed in polyphenolic or flavonoid-rich plant extracts. Similar pro-apoptotic effects have been reported for other Myrtaceae family members, where bioactive phenolics and terpenoids contribute to cytotoxicity by inducing cell cycle arrest and apoptosis in breast and colorectal carcinoma models [59]. The higher apoptotic response in MCF-7 cells (31.41 ± 1.87%) compared to Caco-2 cells (22.37 ± 0.16%) may be attributed to differences in cell-type sensitivity, receptor expression, or intrinsic apoptotic pathway regulation. These results align with the MTT cytotoxicity assay, which demonstrated dose-dependent reduction of cell viability, and with the cell cycle analysis(data discussed in the previous section). Overall, these findings support the antiproliferative and pro-apoptotic potential of *C. macropunctatus* fruit extract, suggesting its bioactive constituents could contribute to natural anticancer drug development. Further studies, including caspase activity assays, mitochondrial membrane potential analysis, and in-vivo validation, are warranted to elucidate the exact molecular mechanisms underlying apoptosis induction.

**Table 4:** Annexin V-FITC/PI apoptosis analysis of *C. macropunctatus* fruit ethanol extract and its impact on MCF-7 and Caco-2

Cell line	Treatment	Early apoptosis %	Late apoptosis %	**Total apoptosis %**	**Necrosis**
MCF7	Control	0.27 ± 0.05	0.18 ± 0.03	1.61 ± 0.12	1.16 ± 0.10
MCF7	Ethanolic extract	19.21 ± 1.35*	7.55 ± 0.62*	31.41 ± 1.87*	4.65 ± 0.35*
Caco-2	Control	0.44 ± 0.88	0.13 ± 0.02	2.16 ± 0.18	1.59 ± 0.12
Caco-2	Ethanolic extract	12.16 ± 0.96*	7.57 ± 0.54*	22.37 ± 0.16*	2.64 ± 0.21*

Results are expressed as mean ± SD. n= 3 independent experiments; Student’s t-test was used for statistical analysis and * indicates statistically significant against control on each stage at *p* < 0.05

The progression of the cell cycle depends on cyclin-dependent kinases (CDK), which are a set of regulatory complexes responsible for phosphorylating substrates involved in the cell cycle. CDK6 functions as the catalytic component of the CDK6-cyclin D complex, which plays a crucial role in the transition from the G1 to S phase of the cell cycle [60]. The activity of the enzyme emerges at the middle of the G1 phase to add a phosphate group. Recent findings indicate that specific cancer cells rely on CDK6 for their growth and division. Therefore, CDK6 is a highly attractive target for the development of anti-cancer treatments [61].

**Fig. 7:**
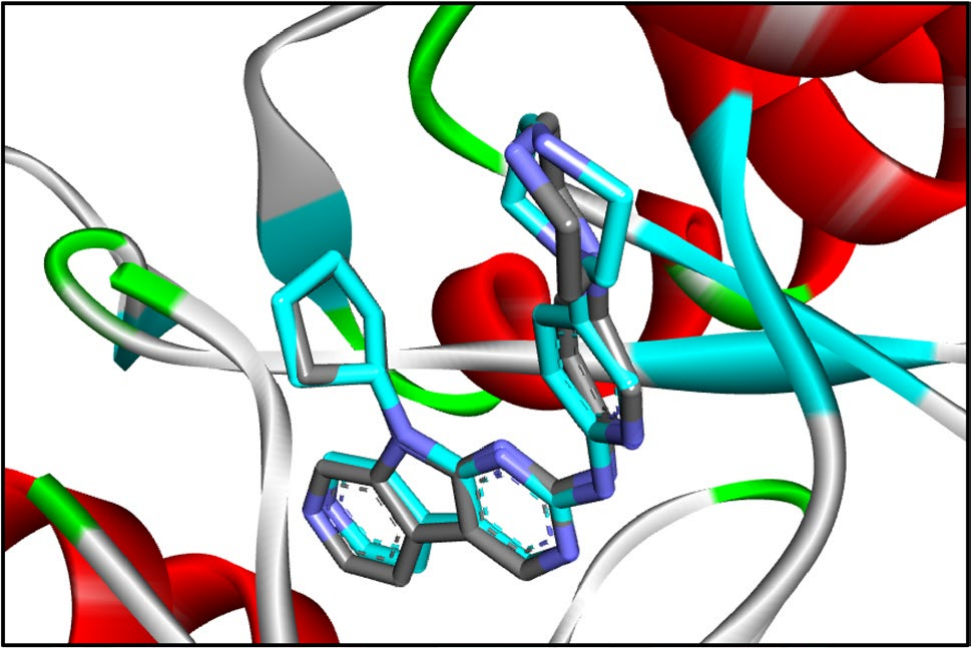
The root mean square deviation (RMSD) between the original and docked poses of the co-crystal ligands for the CDK6 enzyme (PDB: 4tth) was 0.38 Å

The sixteen compounds used in molecular docking of the current study were selected from the metabolites identified through UPLC–ESI–MS/MS profiling of the ethanolic fruit extracts of *Callistemon* species. These phytochemicals were chosen based on their tantatively identification and represent major secondary metabolite classes detected in the four extracts, including phenolic acids, flavonols, lignans, β-triketones, acylphloroglucinols, terpenoids, and meroterpenoids. Additionally, all the 16 compouds were reported to be isolated from different *Callistemon* species belonging to family Myrtaceae. As a result of the molecular docking study, the majority of the identified compounds in the *Callistemon* fruit extracts, were accurately bound to the active sites of the CDK6 enzyme. This is demonstrated in Table 5 and Table S2 (Supplementary data). The enzyme performed re-docking with the co-crystallized ligand to confirm its binding affinity and conformation. The efficacy of the docking methods was showcased by the illustration of a Root Mean Square Deviation (RMSD) value of 0.38Å (Fig. 7). The analysed compounds showed docking scores ranging from (−6.5 to −9.7 kcal/mol) when tested against CDK6. In comparison, the cocrystal ligand had a docking value of −9.1 kcal/mol. The additional data includes Table S1. A total of 16 molecules demonstrates a docking energy that is inferior to that of the cocrystal ligand. The phenolic compounds avicularin, nilocitin, and quercetin 3-*O*-(2’’-galloyl)-*β*-D-galactopyranoside exhibited the maximum stability when docked, as evidenced by docking scores greater than − 9.5 kcal/mol.

**Table 5:** Docking results of the analyzed compounds againstCDK6 enzyme active site

Compound name	Amino acid/Interaction/Distance Å/E(kcal/mol)	Energy scores (kcal/mol)
Co-crystallized ligand	Val101/H-Bond/2.33 Asp163/H-Bond/2.86 Val101/H-Bond/3.07 Glu99/H-C-Bond/3.55 Asp104/Pi-Anion/3.85 Ile19/Pi-Sigma/3.80 Leu152/Pi-Sigma/3.57 Leu152/Pi-Sigma/3.31 Ala162/Pi-Sigma/3.76 Val27/Alkyl/4.72 Ala41/Pi-Alkyl/5.16 Val77/Pi-Alkyl/5.16 Val27/Pi-Alkyl/5.23 Ala41/Pi-Alkyl/4.69 Ala162/Pi-Alkyl/4.53 Ile19/Pi-Alkyl/5.15 Ala41/Pi-Alkyl/4.36 Val101/Pi-Alkyl/5.11	−9.1
Avicularin	Lys43/H-Bond/2.60 Asp104/H-Bond/2.37 Val101/H-Bond/3.00 Asp104/H-Bond/3.32 Asp163/H-Bond/3.18 Ile19/H-Bond/3.34 Gly20/H-C-Bond/3.15 Phe98/Pi-Donor H- Bond/3.16 Ala162/Pi-Sigma3.80 Ile19/Pi-Alkyl/4.23 Leu152/Pi-Alkyl/4.91 Val27/Pi-Alkyl/4.21 Ala162/Pi-Alkyl/4.78 Val27/Pi-Alkyl/5.17	−9.7
Nilocitin	Lys43/H-Bond/2.41 Val101/H-Bond/1.92 Asp104/H-Bond/2.64 Lys147/H-Bond/2.48 Ile19/H-Bond/3.19 Glu61/H-Bond/2.67 Val101/H-Bond/2.93 Asp102/H-Bond/3.29 Val101/H-Bond/2.75 Gly20/C- H-Bond/3.63 His100/Pi-Donor H-Bond/3.55 Ile19/Pi-Sigma/3.61 Val27/Pi-Alkyl/5.46 Leu152/Pi-Alkyl/4.52	−9.6
quercetin 3-*O*-(2''-galloyl)-*β*-D-galactopyranoside	Lys147/H-Bond/2.61 Gln149/H-Bond/1.90 Gln103/C-H-Bond/3.47 Ile19/C-H-Bond/2.98 Asp104/Pi-Anion/3.99 Phe98/Pi-Donor H-Bond/3.60 Thr106/Pi-Sigma/3.53 Ala162/Pi-Sigma/3.74 Val27/Pi-Alkyl/5.00 Leu152/Pi-Alkyl/4.73 Ala162/Pi-Alkyl/5.09 Val27/Pi-Alkyl/5.08 Leu152/Pi-Alkyl/5.22 Ile19/Pi-Alkyl/3.87	−9.6

clinical animal modelsThe ATP binding site of CDK6 consists of Valine 101, Threonine 106, and Glutamine 149. The catalytic process is facilitated by Glu61, Lys43, and Asp163 residues [58]. The root mean square deviation (RMSD) of 0.38Å was determined by comparing the co-crystallized ligand with the docked ligand (refer to Fig. 7). In the co-crystallized state, the ligand formed a total of five hydrogen bonds with Val101 (at distances of 2.33 Å and 3.07 Å), Asp163 (at distances of 2.86 Å and 3.30 Å), and Glu99 (at a distance of 3.55Å), as well as Pi-Anion bond with Asp104. In addition, it formed Pi-Sigma interactions with Ile19, Leu152, and Ala162, and Alkyl bonds with Val27, Ala41, Val77, Ala162, Ile19, and Val101, as shown in Fig. 8a. Avicularin achieved the highest docking score of −9.7 kcal/mol when tested against CDK6. Avicularin formed a total of eight hydrogen bonds with crucial amino acids in CDK6. The interactions occurred between specific amino acids: Lys43, Asp104, Asp163, Val101, Ile19, Gly20, and Phe98 at a distance ranged from 2.37 Å to 3.34 Å. In addition, it can be demonstrated that the hydrophobic contacts occur with Ala162, Ile19, Leu152, and Val27 as illustrated in Fig. 8b. Conversely, the docking investigation demonstrated that nilocitin displayed a binding pattern that closely resembled that of avicularin. The binding energy exhibited by Nilocitin was − 9.6 Kcal/mol. Nilocitin established a total of ten hydrgen bonds with Lys43, Val101, Asp104, Lys147, Ile19, Glu61, Asp102, Gly20, and His100, as well as three hydrophobic bonds attaches with Ile19, Val27, and Leu152, as illustrated in Fig. 8c. In addition, the molecule quercetin 3-*O*-(2’’-galloyl)-*β*-D-galactopyranoside formed five hydrogen bonds with Gln149, Lys147, Gln103, Ile19, and Phe98 as shown in Fig. 8d. Furthermore, it established nine hydrophobic contacts with Asp104, Thr106, Ala162, Val27, Leu152, Val27, Ala162, and Ile19.

**Fig. 8:**
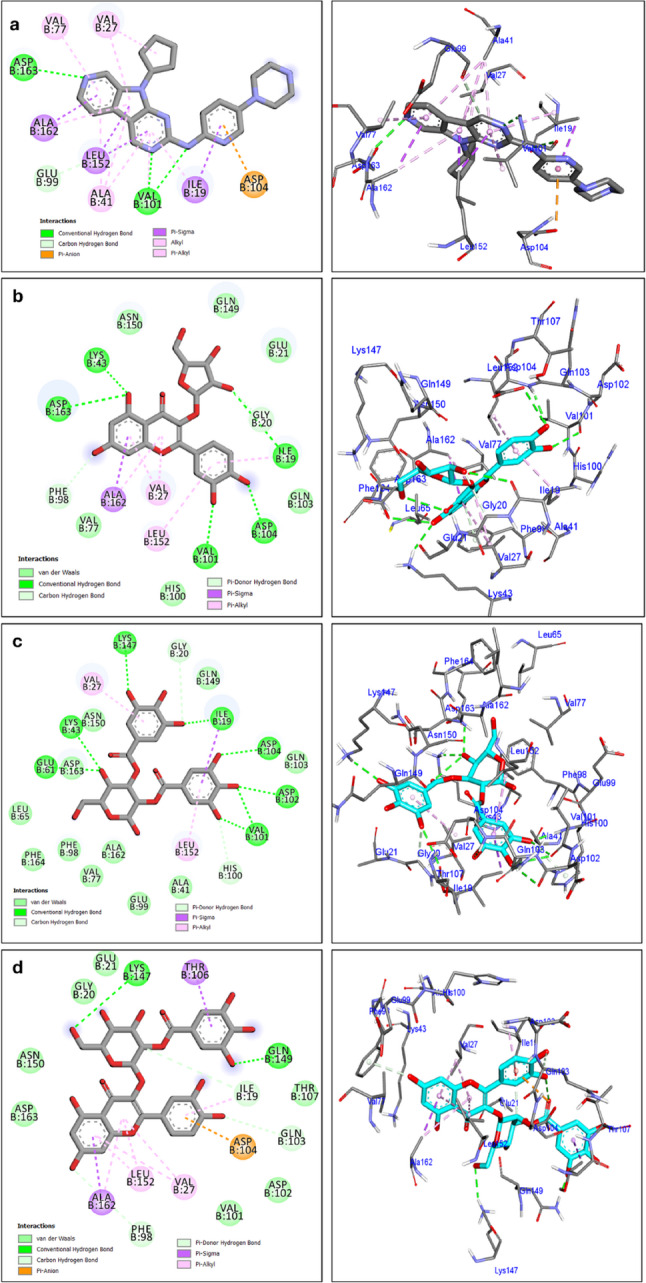
2D and 3D representation of anticipated binding mode for (**a**) Co-crystal ligand, (**b**) avicularin, (**c**) nilocetin, (**d**) quercetin 3-*O*-(2’’-galloyl)-*β*-D-galactopyranoside with CDK6 (PDB: 4tth) active sites

### Pharmacokinetics estimates

Table S1 summarizes the ADMET (absorption, distribution, metabolism, excretion, and toxicity) properties of avicularin, nilocitin, and quercetin 3-O-(2’’-galloyl)-β-D-galactopyranoside. All three compounds violate Lipinski’s Rule of Five, specifically in having more than 10 hydrogen bond acceptors and more than 5 hydrogen bond donors. Additionally, Quercetin 3-O-(2’’-galloyl)-β-D-galactopyranoside presents a molecular weight of 500 g/mol, which is at the upper limit for drug-likeness. The compounds also demonstrated low gastrointestinal (GIT) absorption, suggesting limited oral bioavailability. However, their chemical flexibility, indicated by a number of rotatable bonds ranging from 4 to 7, supports a potential for interaction with diverse biological targets. Importantly, none of the compounds are substrates for P-glycoprotein (P-gp), implying a lower risk of efflux out of cells and potentially enhancing intracellular accumulation and bioactivity. Furthermore, none of the molecules are predicted to cross the blood-brain barrier (BBB), which reduces the risk of central nervous system (CNS) side effects and suggests they may be peripherally selective. From a metabolic perspective, the three compounds did not inhibit key cytochrome P450 isoforms, including CYP1A2, CYP2C19, CYP2C9, CYP2D6, and CYP3A4, indicating a low potential for metabolic drug-drug interactions. Finally, no mutagenic or tumorigenic properties were predicted for any of the compounds, further supporting their favorable safety profile.

## Conclusions

This study investigated the antiproliferative potential of ethanol extracts of four *Callistemon* species (*C. citrinus*, *C. macropunctatus*, *C. viminalis*, and *C. subulatus*) against breast (MCF-7) and colon (Caco-2) cancer cell lines. UPLC-ESI-MS/MS analysis tentatively identified 16 compounds within the extracts, belonging to various chemical classes such as lignans, meroterpenes, and flavonoids. Among all the tested extracts, *C. macropunctatus* exhibited the most potent antiproliferative activity against both cancer cell lines, with IC_50_ values of 5.45 µg/mL for MCF-7 and 10.24 µg/mL for Caco-2. Further investigation revealed that *C. macropunctatus* extract induced cell cycle arrest at the S phase in MCF-7 cells and at both G0/G1 and S phases in Caco-2 cells. Additionally, the extract significantly promoted apoptosis in MCF-7 cells. In silico docking simulations suggested potential mechanisms underlying the antiproliferative activity. Several identified compounds, particularly phenolic compounds like avicularin, nilocitin, and quercetin 3-O-(2’’-galloyl)-*β*-D-galactopyranoside, displayed promising binding affinities towards CDK6, a key cell cycle regulator. These findings warrant further exploration of *C. macropunctatus* extract as a potential source of anti-cancer agents, with a focus on elucidating the role of CDK6 inhibition in its antiproliferative effects. Further isolation and purification of the bioactive compounds identified in *C. macropunctatus* extract, particularly focusing on avicularin, nilocitin, and quercetin 3-O-(2’’-galloyl)-*β*-D-galactopyranoside. Evaluation of the in vivo anti-cancer efficacy of *C. macropunctatus* extract in pre-clinical animal models will be performed.

## Supplementary Information

Supplementary Material 1

## Data Availability

All data generated or analyzed during this study are included in the published article and/or its supplementary information files.

## Abbreviations

CDK: Cyclin-dependent kinase
EGFR: Epidermal growth factor receptor
G1 phase: the first gap
G2 phase: the second gap
HR: Hormone receptor
M phase: Mitosis
MTT: 3-(4,5-dimethylthiazol-2- yl)-2,5-diphenyltetrazolium bromide
PBS: Phosphate-buffered saline
RB: Retinoblastoma
S phase: DNA synthesis
UPLC-ESI-MS/MS: Ultra Performance Liquid Chromatography coupled with mass/mass spectrometry
VEGF: Vascular endothelial growth factor
