# Supplementary material for: Unveiling the cytotoxic potential of four Callistemon fruit extracts against breast and colon cancer: a combined metabolomic and in silico approach
Source: BMC Complement Med Ther. 2026 Jan 15;26:29. doi: 10.1186/s12906-025-05224-y (PMC12849428; doi:10.1186/s12906-025-05224-y)
Supplement: Supplementary file 1 — Supplementary Material 1 [file 12906_2025_5224_MOESM1_ESM.docx]

**Unveiling the Antitumor Potential of Callistemon Fruit Extracts in Breast and Colon Cancer: A Combined Metabolomic and In Silico Approach**

Amira Y. Eissa^1^, Kamilia F. Taha^1^, Abeer Dahab^2^, Usama R. Abdelmohsen^3,4^, Khayreya A. Youssif^5^, Mona H. Ibrahim^6^, Seham S. El-Hawary^7^, Manal M. Sabry^7,^*


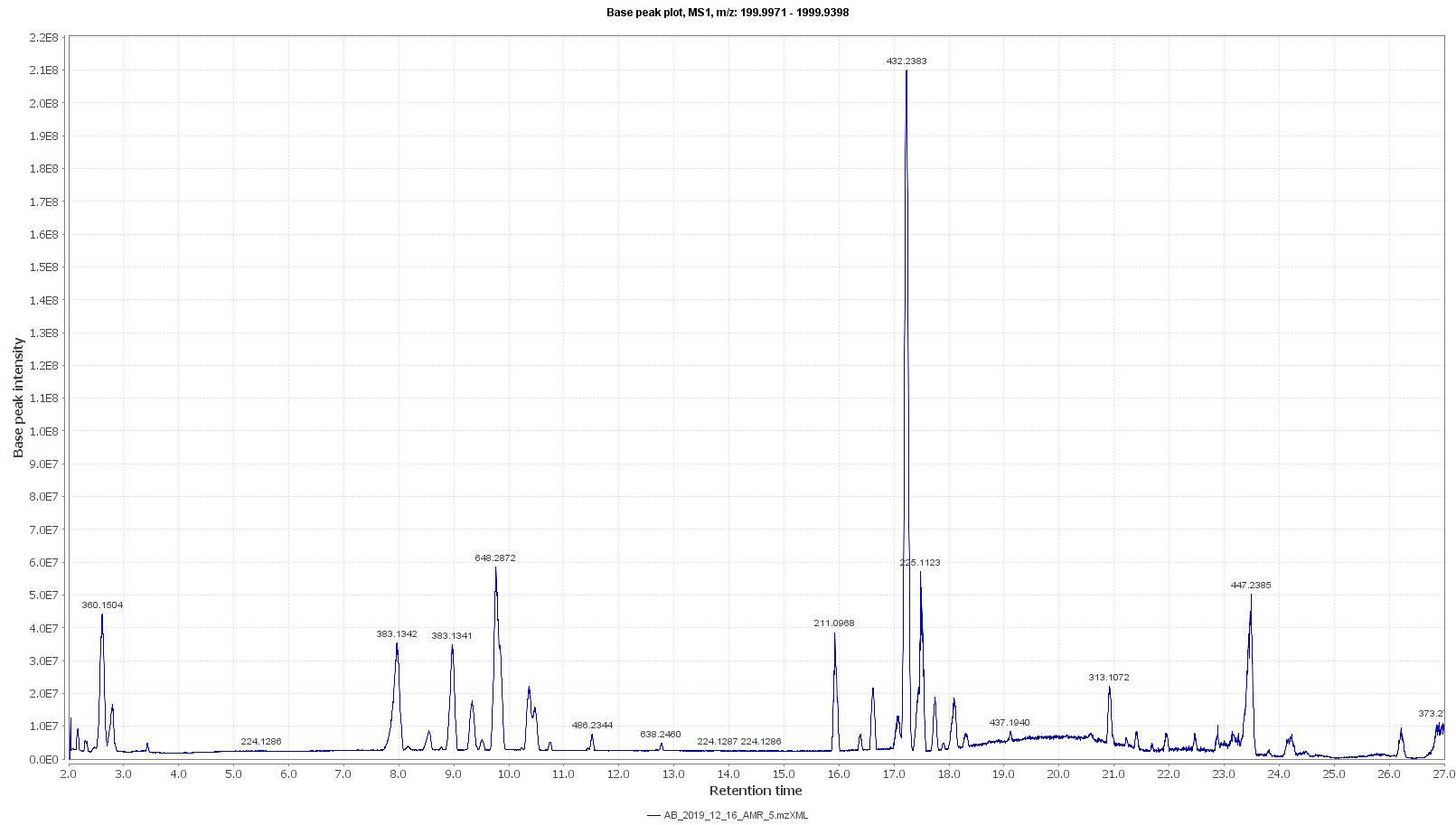


**Fig. S1: Total LC/MS chromatogram of ethanolic fruit extract of *Callistemon citrinus***


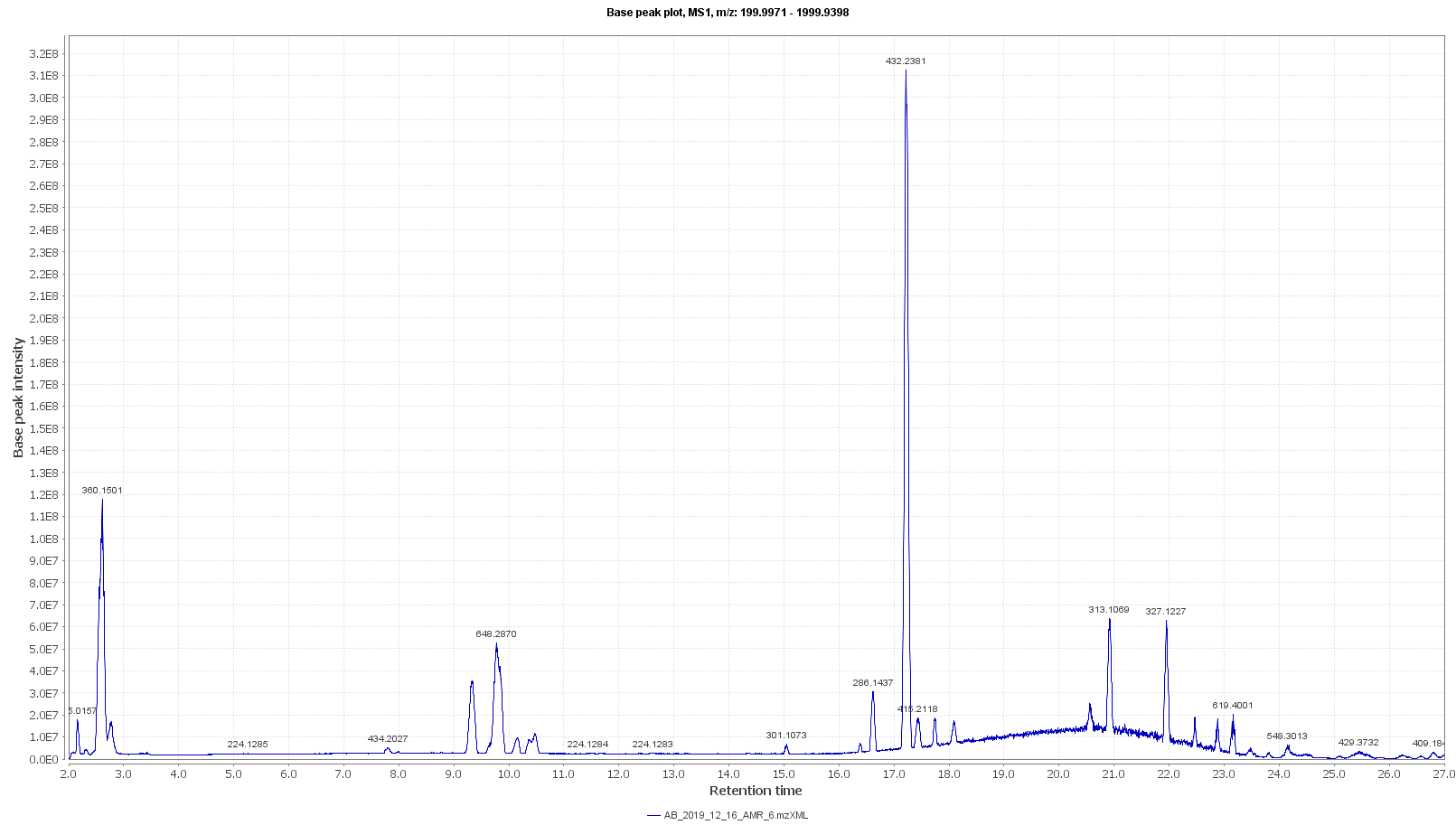


**Fig. S2: Total LC/MS chromatogram of ethanolic fruit extract of *Callistemon macropunctatus***


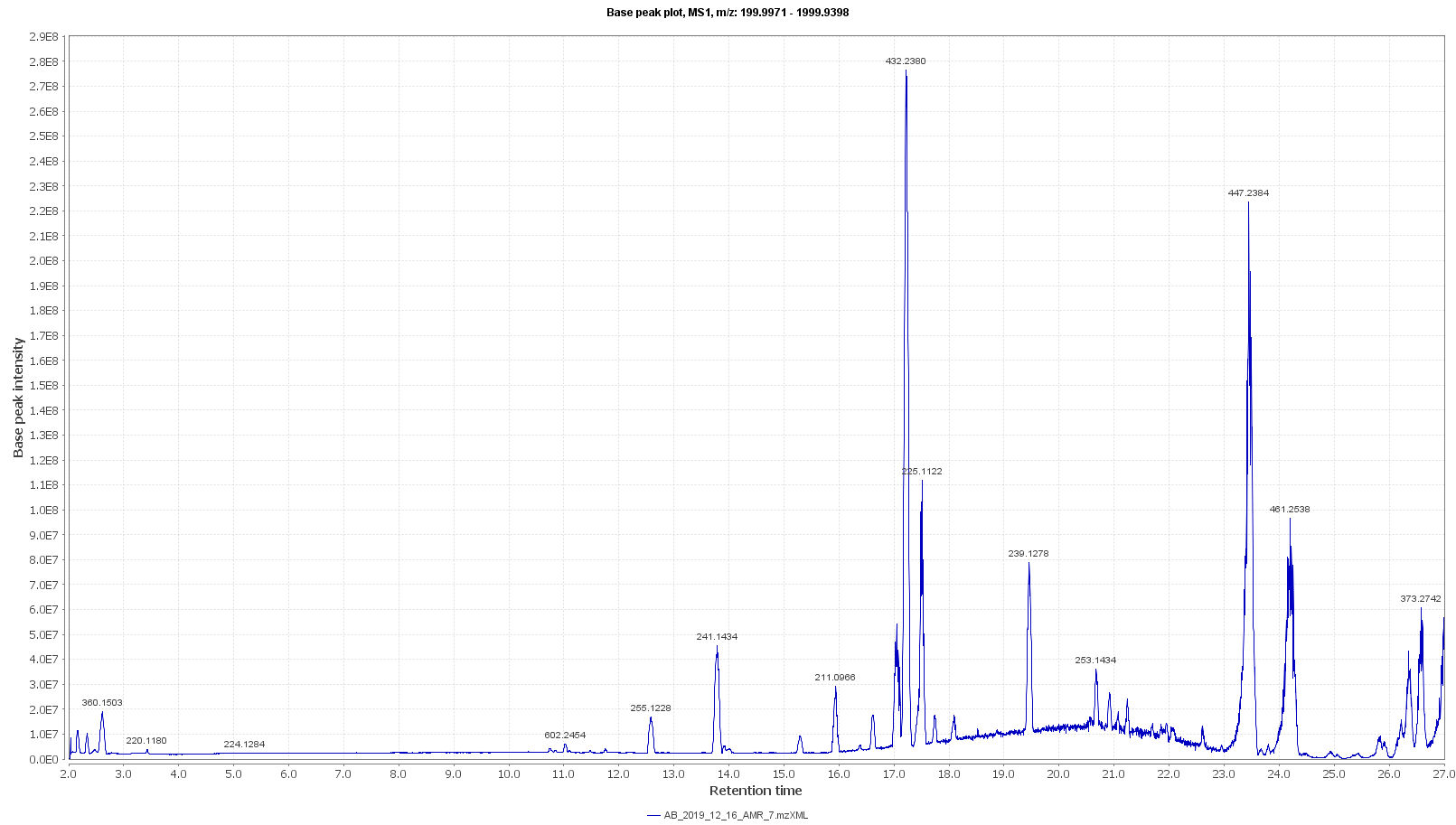


**Fig. S3: Total LC/MS chromatogram of ethanolic fruit extract of *Callistemon subulatus***


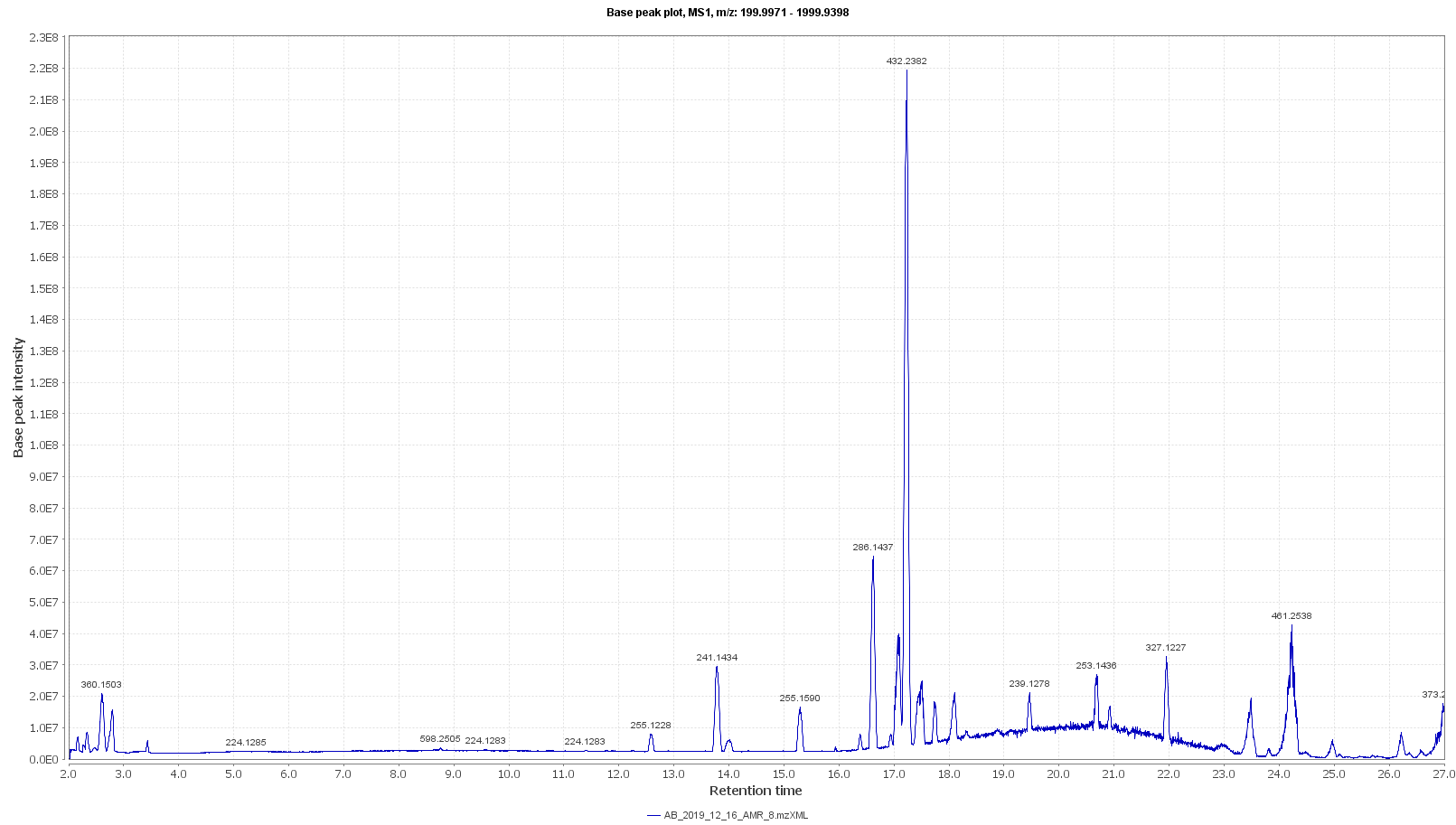


**Fig. S4: Total LC/MS chromatogram of ethanolic fruit extract of *Callistemon viminalis***

| **Co.** | Avicularin | Nilocitin | Quercetin 3-O-(2''-galloyl)-beta-D-galactopyranoside |
| --- | --- | --- | --- |
| **MW** | 434.35 | 484.36 | 616.48 |
| **H-acceptor** | 11 | 14 | 16 |
| **H-donor** | 7 | 9 | 10 |
| **MLOGP** | -2.06 | -2.3 | -2.77 |
| **Lipinski violations** | 2 | 2 | 3 |
| **No. of Rotatable bond** | 4 | 7 | 7 |
| **GI absorption.** | Low | Low | Low |
| **BBB perm.** | No | No | No |
| **Pgp subst.** | No | No | No |
| \| **CYP1A2 inhibitor** \|  \|  \|  \|  \| \| --- \| --- \| --- \| --- \| --- \| | No | No | No |
| **CYP2C19 inhibitor** | No | No | No |
| **CYP2C9 inhibitor** | No | No | No |
| **CYP2D6 inhibitor** | No | No | No |
| **CYP3A4 inhibitor** | No | No | No |
| **Mutagenicity** | None | None | None |
| **tumorigenicity** | None | None | None |

**Table S1:** the ADMT of Avicularin, Nilocitin, and Quercetin 3-O-(2''-galloyl)-beta-D-galactopyranoside.

**Table S2:** Docking energy scores of **16 compounds** with in CDK6

| No. | Compound name | **Docking energy scores in kcal/mol** |
| --- | --- | --- |
|  | Ligand | -9.1 |
| 1 | 3-Epiursolic acid | -6.7 |
| 2 | Alphitolic acid | -8.3 |
| 3 | Astragalin | -9.5 |
| 4 | Avicularin | -9.7 |
| 5 | Callisalignone C | -7.6 |
| 6 | Callislignan B | -9.1 |
| 7 | Callistrilone O | -6.5 |
| 8 | Callistrilones A | -7.6 |
| 9 | Calliviminol B | -8.3 |
| 10 | Calliviminones G | -8.1 |
| 11 | Myrtucommulone B | -8.3 |
| 12 | Nilocitin | -9.6 |
| 13 | Quercetin 3-O-(2''-galloyl)-beta-D-galactopyranoside | -9.6 |
| 14 | Quercetin | -9.5 |
| 15 | Viminalin I | -9.3 |
| 16 | Viminalin O | -8.6 |
